# Primary motor hand area corticospinal excitability indicates overall functional recovery after spinal cord injury

**DOI:** 10.3389/fneur.2023.1175078

**Published:** 2023-06-02

**Authors:** Chun-Qiu Dai, Ming Gao, Xiao-Dong Lin, Bai-Jie Xue, Ying Liang, Mu-Lan Xu, Xiang-Bo Wu, Gui-Qing Cheng, Xu Hu, Chen-Guang Zhao, Hua Yuan, Xiao-Long Sun

**Affiliations:** ^1^Department of Rehabilitation Medicine, Xi-Jing Hospital, Air Force Medical University (Fourth Military Medical University), Xi’an, China; ^2^Lintong Rehabilitation and Convalescent Centre, Xi'an, China; ^3^Department of Health Statistics, Air Force Medical University (Fourth Military Medical University), Xi’an, China

**Keywords:** spinal cord injury, corticospinal excitability, MEP, ADL, motor function

## Abstract

**Background:**

After spinal cord injury (SCI), the excitability of the primary motor cortex (M1) lower extremity area decreases or disappears. A recent study reported that the M1 hand area of the SCI patient encodes the activity information of both the upper and lower extremities. However, the characteristics of the M1 hand area corticospinal excitability (CSE) changes after SCI and its correlation with extremities motor function are still unknown.

**Methods:**

A retrospective study was conducted on the data of 347 SCI patients and 80 healthy controls on motor evoked potentials (MEP, reflection of CSE), extremity motor function, and activities of daily living (ADL) ability. Correlation analysis and multiple linear regression analysis were conducted to analyze the relationship between the degree of MEP hemispheric conversion and extremity motor function/ADL ability.

**Results:**

The CSE of the dominant hemisphere M1 hand area decreased in SCI patients. In 0–6 m, AIS A grade, or non-cervical injury SCI patients, the degree of M1 hand area MEP hemispheric conversion was positively correlated with total motor score, lower extremity motor score (LEMS), and ADL ability. Multiple linear regression analysis further confirmed the contribution of MEP hemispheric conversion degree in ADL changes as an independent factor.

**Conclusion:**

The closer the degree of M1 hand area MEP hemispheric conversion is to that of healthy controls, the better the extremity motor function/ADL ability patients achieve. Based on the law of this phenomenon, targeted intervention to regulate the excitability of bilateral M1 hand areas might be a novel strategy for SCI overall functional recovery.

**Figure:**
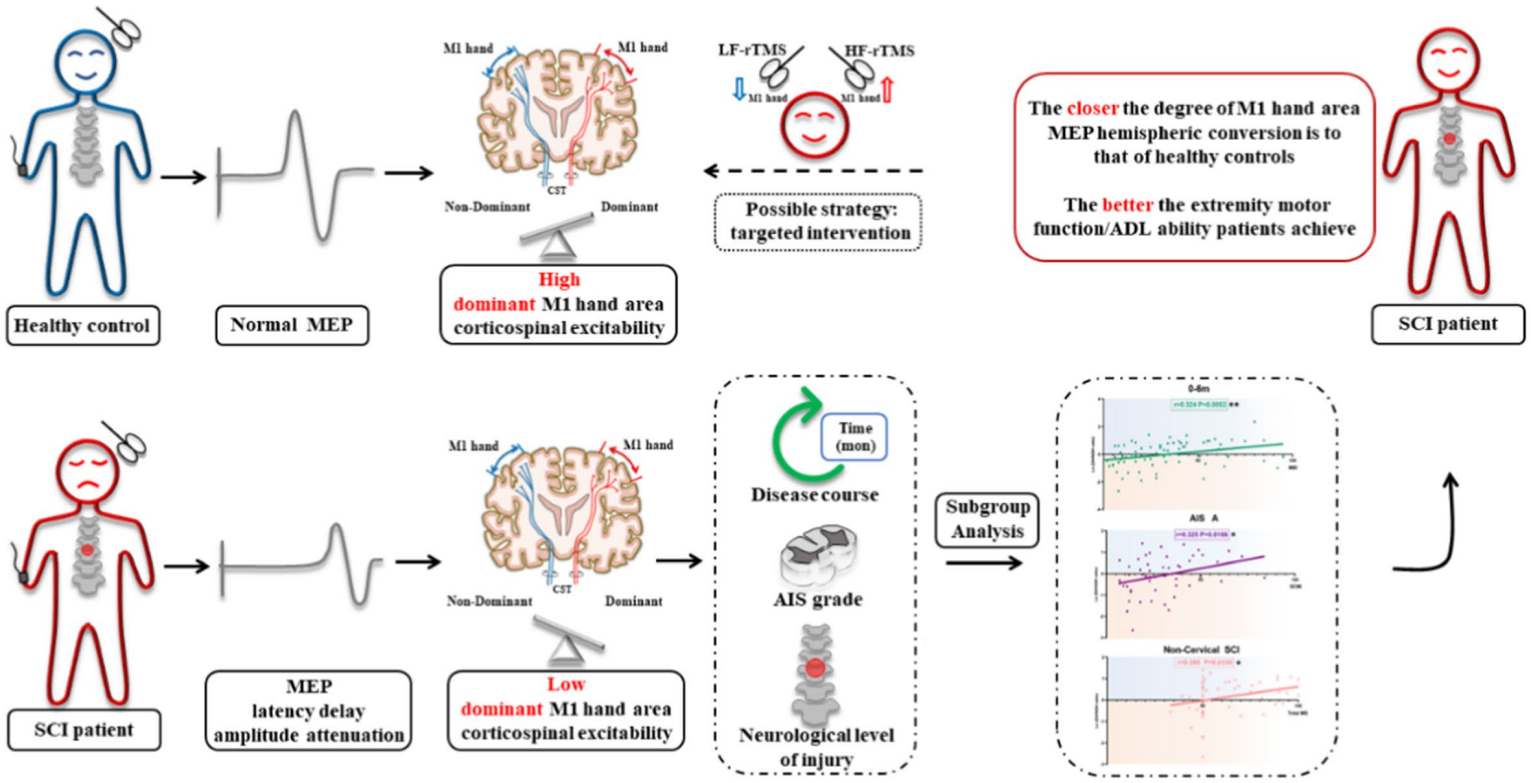
Graphical abstract.

## 1. Introduction

Spinal cord injury (SCI) can occur often in young adults, and the improvement of clinical first aid has resulted in more surviving patients who unfortunately suffer from severe motor dysfunction (MD) of the extremity below the injured neurological level (1, 2).

Voluntary movement of the extremity is dominated mainly by the primary motor cortex (M1). Although the site of injury in SCI is in the spinal cord, significant structural remodeling and electrophysiological changes can also occur in the M1 area, manifesting as a decrease or disappearance of excitability in the M1 lower extremity (LE) area (3, 4). Unfortunately, the efficacy of intervention strategy toward M1 LE area was only moderate. Previous research found that, even after 4-week of repeated transcranial magnetic stimulation (rTMS) treatment toward the LE area, the gait function of SCI patients did not significantly improve when compared with a sham stimulation group (5). A possible reason for this may be due to the M1 LE area’s deep location, small area, and the limited efficacy of the current figure-8, circular, and even double-cone rTMS coils (6). Thus, there is still no recommendation for MD after SCI in the latest rTMS guideline published in 2020 (7).

Interestingly, a recent study reported that the hand area of the central anterior gyrus (the location of the M1 area in the traditional sense, which the author explained as the premotor area in the article) of an SCI patient encoded the activity information of both the upper extremity (UE) and LE, and the movement coding of the UE and LE were highly correlated (8). The above study suggested that the M1 hand area may also encode LE activity in SCI patients. The M1 hand area is large and shallow, which is accepted as relatively excellent stimulation area in clinic. Thus, targeted regulation on the excitability of the M1 hand area of SCI patients may be able to effectively promote the recovery of UE and LE movement. Some researches have unexpectedly observed that M1 hand area rTMS treatment may restore LE motor function. Belci et al. (9) and Gunduz et al. (10) revealed that high frequency rTMS stimulation on the left M1 hand area in patients with incomplete SCI, resulted in significant improvement of overall motor function. However, the underlying mechanism is largely unknown. In view of these facts, changes in the excitability of the bilateral M1 hand area and its correlation with extremities motor function after SCI are urgently required to improve the therapeutic effect of rTMS-targeted stimulation.

Motor evoked potentials (MEP) elicited by TMS is the preferable quantification toward the excitability of elements controlling motor output, including the M1 area and corticospinal tract (CST), providing the evaluation of corticospinal excitability (11). A research found that the prognostic accuracy of MEP appears much higher than that of clinical examination for stoke patients, indicating the predictive function of MEP (12). Sheng W et al. demonstrated that the increased muscle co-contraction was associated with the CST excitability impairment assessed by MEP in stroke survivors, and improvement in CST may contribute to the recovery of muscle discoordination (13).

Therefore, we herein set out to observe the MEP changes of bilateral M1 hand area after SCI and its correlation with extremities motor function and ADL ability with the ultimate goal of providing novel targets and ideas for improving the effect of clinical rTMS treatment in SCI.

## 2. Methods

### 2.1. Study design

This cross-sectional retrospective study included neurophysiologic and motor functional data of SCI patients and healthy controls from January 2017 to August 2022 that were collected at the inpatient unit of the Department of Rehabilitation Medicine in Xijing Hospital.

This study was approved by the Ethics Review committee of the Xijing Hospital (No. KY20222073-C-1). Informed consent is exempted due to the characteristics of retrospective study.

### 2.2. Participants

SCI patient inclusion criteria: a. age ≥ 18 years old, right hand dominant (Edinburgh Handedness Inventory); b. has been diagnosed with SCI; c. no cognitive impairment (Mini-Mental State Examination≥24 scores) (14); d. disease course≤36 months (from SCI to clinical assessment and MEP measurement, patient has tided over the spinal shock). Exclusion criteria: a. UE fracture with peripheral nerve injury; b. severe coagulation disorder, severe cardiac insufficiency, or uncontrollable hypertension; c. other serious systemic diseases such as tumor; d. those taking any drug that may affect the MEP recording within 1 week, including baclofen, diazepam, and other drugs (15); e. absolute contraindication for TMS examination, such as ferromagnetic metal or microprocessor implants in the head (plates, shrapnel, or cochlear implants) (16); f. refuse M1 hand area MEP measurement.

Healthy control inclusion criteria: age ≥ 18 years old, right hand dominant. Exclusion criteria included the above situations that are not available for MEP recording.

### 2.3. Electrophysiological recordings

MEPs were recorded from the abductor pollicis brevis muscle (17) through surface Ag-AgCl electrodes (10 mm diameters) placed at the belly-tendon montage using TMS equipment (MEP-9404C, Japan). A single pulse TMS was delivered to the hotspot under 80% of the maximum stimulator output (18). MEPs were recorded for a 5 s interval.

### 2.4. Hemispheric CSE evaluation of bilateral M1 hand areas

Referring to the previously reported methods evaluating the hemispheric CSE changes, we then evaluated the hemispheric CSE conversion degree of the bilateral M1 hand areas and calculated the conversion degree as ln (dominant/non-dominant hemisphere M1 hand area MEP amplitude) (19). A value of 0 indicated that excitability of the dominant hemisphere (DH) was equal to that of the non-dominant hemisphere (NDH) [ln (1)=0], while >0 indicated that excitability of the DH was higher than that of the NDH [ln (>1) > 0], and < 0 indicated that the excitability of the DH was lower than that of the NDH [ln (<1) < 0].

### 2.5. Clinical assessment

#### 2.5.1. Motor score

MS mainly evaluates the muscle strength of key muscles innervated by different spinal nerves (20). The total MS is 100 which consists of UEMS and LEMS, with 50 points each.

#### 2.5.2. Modified Ashworth scale

MAS was used to measure the muscle tone (21). The classification standard includes grades 0–4 (0, 1, 1+, 2, 3, 4), that are transferred into scores from 0 to 5 in analysis.

#### 2.5.3. Modified Barthel index

MBI (22) is the most commonly used scale for evaluating overall ADL ability, which includes 10 items. The total score is 100.

#### 2.5.4. Spinal cord independence measure

SCIM was used to evaluate the prognosis and ADL ability of SCI patients (23). The total score is 100. There are 17 items in total, of which item 12 (SCIM12) (24) is of great significance for the evaluation of indoor mobility function of SCI patients. The total score for SCIM12 is 8.

### 2.6. Statistical analysis

#### 2.6.1. Descriptive statistics

Measurement or enumeration data are presented as the mean ± SEM (standard error of the mean) or number (%) [N (%)]. The *t-test*, *Chi-squared test*, or *Fisher’s exact test* were utilized for data comparison. *p < 0.05 was considered significantly different.

#### 2.6.2. MEP and hemispheric conversion degree

All data were analyzed by *normality test* before analysis. On the basis of *Kolmogorov–Smirnov test*, the data of MEP amplitude was not normally distributed, therefore, the MEP amplitudes of patients and controls were analyzed by *non-parametric test* (25). The MEP latency and central motor conduction time (CMCT) were analyzed with *t-test* of independent samples. The hemispheric conversion degree[ln (DH/NDH ratio)] was compared with *one-way ANOVA* with the factor of disease course (control, 0–6 m, 6–36 m). *p < 0.05 was considered significantly different.

#### 2.6.3. Correlation analysis and multiple linear regression analysis

Correlation analysis of the M1 hand area MEP amplitude and motor function/ADL ability were investigated for evaluating the effects of disease course, American Spinal Injury Association impairment scale (AIS), and neurological level of injury (NLI). Pearson correlations between different phases/groups were calculated after *normality test*.

Univariate and multivariate linear regression analyses were conducted to validate the influence of M1 hand area MEP hemispheric conversion degree as the independent factor. MBI and SCIM were set as the linear regression analysis outcomes. The univariate analysis conducted the inclusion criteria as *p <* 0.10.

*p < 0.05 was considered significantly different.

## 3. Results

### 3.1. Basic characteristics of SCI patients and healthy controls

**Figure 1:**
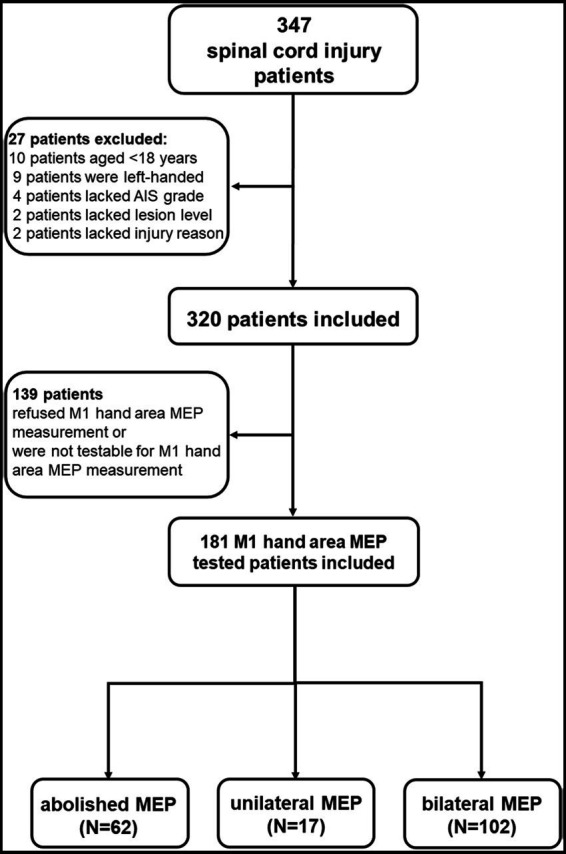
Flow chart of this study. The data of 347 SCI patients were collected from 2017 and 2022 in the inpatient unit of the Department of Rehabilitation Medicine of Xijing Hospital. Twenty-seven SCI patients were excluded because they failed to meet the inclusion criteria, and 139 patients refused M1 hand area MEP measurement or were not testable for M1 hand area MEP measurement due to the exclusion criteria. The included 181 patients were divided into three groups which included: a) M1 hand area MEP could not be measured (abolished MEP, aMEP, 62 patients), b) unilateral M1 hand area MEP could be measured (unilateral MEP, uniMEP, 17 patients), c) bilateral M1 hand area MEP could be measured (bilateral MEP, biMEP, 102 patients). Meanwhile, nine healthy controls were excluded due to the exclusion criteria, and the data of 71 right-handed healthy controls were analyzed in comparison with the biMEP group.

**Figure 2:**
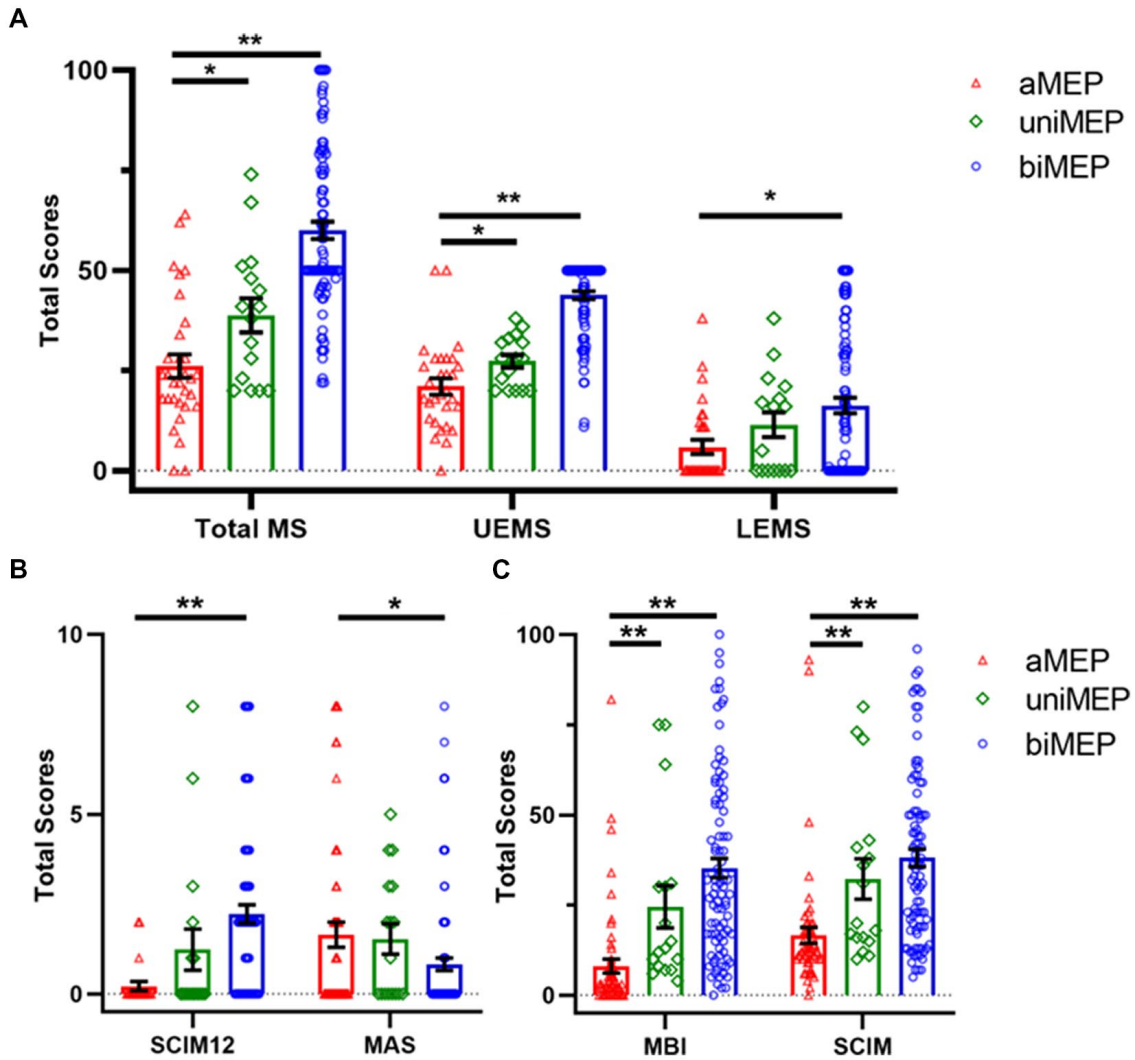
Comparison of extremity motor function and ADL ability in the tested M1 hand area MEP subgroup. **(A)** Comparison of motor score in the tested M1 hand area MEP subgroup. The total MS, UEMS, and LEMS showed an upward trend from aMEP, uniMEP, to biMEP, sequentially. **(B)** Comparison of SCIM12 and MAS in the tested M1 hand area MEP subgroup. Results of SCIM12 showed an upward trend from aMEP, uniMEP, to biMEP, sequentially. MAS showed a downward trend from aMEP, uniMEP, to biMEP, sequentially. **(C)** Comparison of MBI and SCIM in tested M1 hand area MEP subgroup. Results indicated that MBI and SCIM showed an upward trend from aMEP, uniMEP to biMEP, sequentially. *p<0.05, **p<0.01. ADL: activities of daily living; aMEP: abolished MEP group; uniMEP: unilateral MEP group; biMEP: bilateral MEP group; MEP: motor evoked potentials; UEMS: upper extremity motor score; LEMS: lower extremity motor score; M1 hand area MEP: upper extremity MEP; SCIM: spinal cord independence measure; MBI: modified Barthel index; MAS: modified Ashworth scale.

Data from 347 SCI patients and 80 healthy controls were collected. Twenty-seven SCI patients were excluded due to the inclusion criteria, and 139 patients refused M1 hand area MEP measurement or were not testable for M1 hand area MEP measurement due to the exclusion criteria. The final included 181 patients were divided into three groups: (a) M1 hand area MEP could not be measured (abolished MEP, aMEP, *n* = 62) (26), (b) unilateral M1 hand area MEP could be measured (unilateral MEP, uniMEP, *n* = 17), and (c) bilateral M1 hand area MEP could be measured (bilateral MEP, biMEP, *n* = 102) (Figure 1). Meanwhile, nine healthy controls were excluded due to the exclusion criteria, and data from 71 right-handed healthy controls were analyzed. The basic characteristics of untested M1 hand area MEP and tested M1 hand area MEP SCI patients are shown in Table 1. The basic characteristics of the aMEP, uniMEP, and biMEP SCI groups are shown in Table 2. The basic characteristics of the healthy controls included age in years (39.1 ± 1.2) and gender [male N (%): 54 (76.1%)], with no significant difference compared with the biMEP SCI patients. Motor function/ADL ability among the tested M1 hand area MEP subgroups were further compared. Results indicated that the measurability of M1 hand area MEP was in parallel with motor function and ADL ability, biMEP patients had higher extremity motor function/ADL scores (Figure 2).

**Table 1:** Basic characteristics of untested MEP and tested MEP SCI patients.

	Untested MEP	Tested MEP	*t*/*χ*^2^	*P*
Number of patients (N)	139	181	–	–
Age in years (Mean ± SE)	38.5 ± 1.2	41.0 ± 1.0	0.696	0.111
Gender [N (%)]				
Female	32 (23.0)	38 (21.0)	0.189	0.664
Male	107 (77.0)	143 (79.0)		
Disease course				
0–6 m	96 (69.1)	140 (77.3)	2.787	0.095
6–36 m	43 (30.9)	41 (22.7)		
NLI [N (%)]				
C	13 (9.4)	118 (65.2)	106.655	**<0.001**
T	59 (42.4)	40 (22.1)		
L	59 (42.4)	18 (9.9)		
S	8 (5.8)	5 (2.8)		
AIS grade [N (%)]				
A	57 (41.0)	86 (47.5)	12.659	**0.005**
B	12 (8.6)	35 (19.3)		
C	29 (20.9)	26 (14.4)		
D	41 (29.5)	34 (18.8)		

SCI, spinal cord injury; MEP, motor evoked potentials; AIS, American Spinal Injury Association (ASIA) impairment scale; NLI, neurological level of injury. Bold values indicates statistical differences of *p*-value.

**Table 2:** Basic characteristics of tested M1 hand area MEP SCI subgroups patients.

	aMEP	uniMEP	biMEP	LSD-t/Fisher’s	*P*
Number of subjects (N)	62	17	102	–	–
Age in years (Mean ± SE)	40.3 ± 1.7	43.7 ± 3.2	40.9 ± 1.4	–	>0.050
Gender [N (%)]					
Female	13 (21.0)	4 (23.5)	21 (20.6)	0.202	0.963
Male	49 (79.0)	13 (76.5)	81 (79.4)		
Disease course					
0–6 m	51 (82.3)	9 (52.9)	80 (78.4)	6.029	>0.050
6–36 m	11 (17.7)	8 (47.1)	22 (21.6)		
NLI [N (%)]					
C	62 (100)	17 (100)	39 (38.2)	83.188	**<0.001**
T	0	0	40 (39.2)		
L	0	0	18 (17.6)		
S	0	0	5 (5.0)		
AIS grade [N (%)]					
A	32 (51.6)	0	54 (52.9)	60.224	**<0.001**
B	21 (33.9)	7 (41.2)	7 (6.9)		
C	9 (14.5)	4 (23.6)	13 (12.7)		
D	0	6 (35.2)	28 (27.5)		

SCI, spinal cord injury; AIS, American Spinal Injury Association (ASIA) impairment scale; NLI, neurological level of injury; aMEP, abolished MEP group; uniMEP, unilateral MEP group; biMEP, bilateral MEP group. Bold values indicates statistical differences of *p*-value.

### 3.2. The CSE of the DH M1 hand area decreased in SCI patients

**Figure 3:**
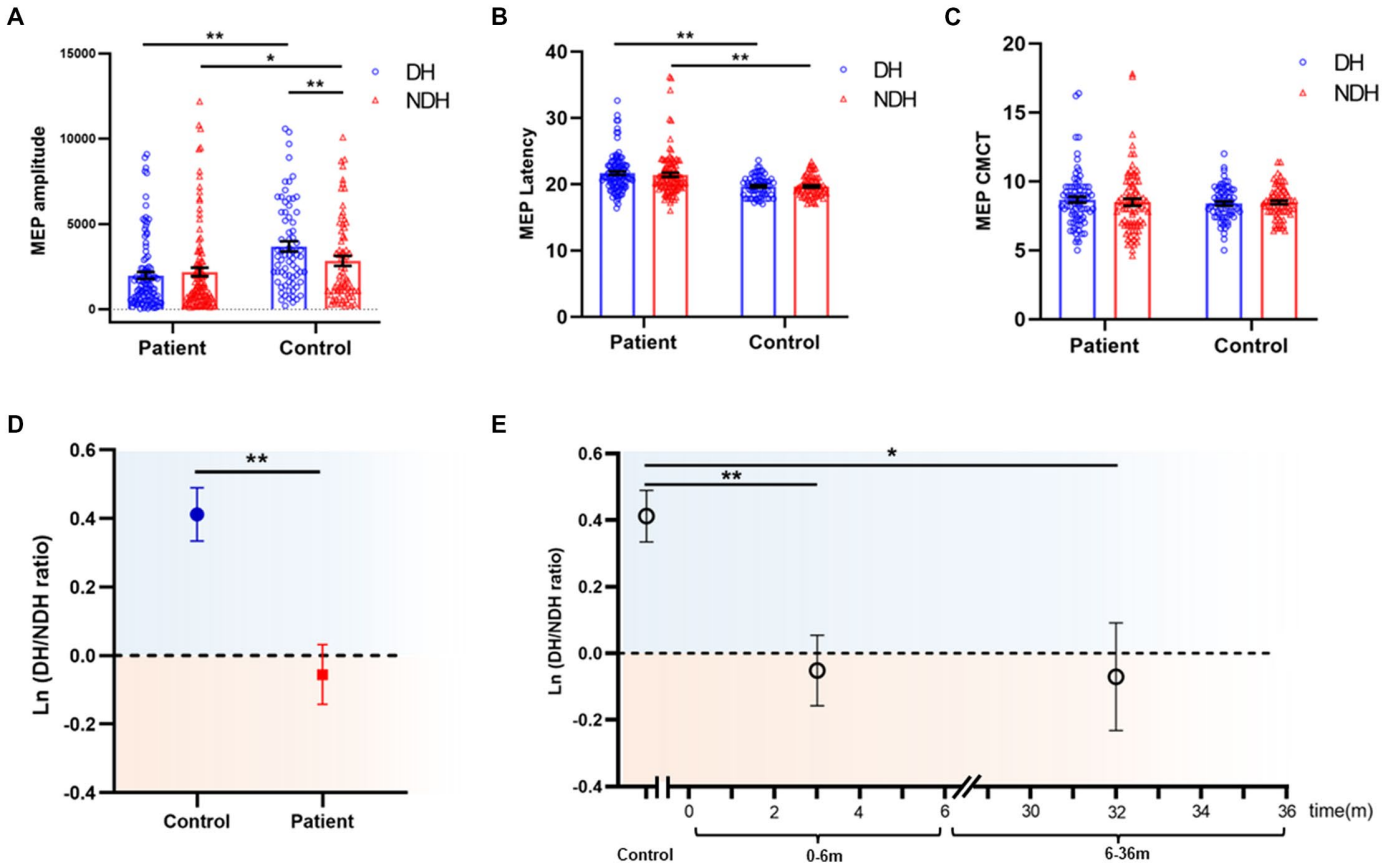
M1 hand area MEP comparison of biMEP SCI patients and healthy controls. **(A)** Comparison of M1 hand area MEP amplitude of biMEP SCI patients and healthy controls. In healthy controls, the DH M1 hand area MEP amplitude was larger than the NDH M1 hand area MEP amplitude. In SCI patients, the opposite phenomenon appeared, in which the NDH M1 hand area MEP amplitude was larger than the DH M1 hand area MEP amplitude. Moreover, the bilateral M1 hand area MEP amplitude in healthy controls was larger than that of SCI patients. **(B)** Comparison of M1 hand area MEP latency of biMEP SCI patients and healthy controls. SCI patients were more delayed than healthy controls in bilateral M1 hand area MEP latency. **(C)** Comparison of M1 hand area MEP CMCT of biMEP SCI patients and healthy controls. No significant difference was found in M1 hand area MEP CMCT. **(D)** The degree of hemispheric CSE conversion was calculated as the ln (dominant / non-dominant hemisphere M1 hand area MEP amplitude). The ln(DH/NDH ratio) was 0.41±0.1 in the controls, but -0.06±0.1 in the SCI patients. The CSE of the DH M1 area decreased in SCI patients compared to the control group. **(E)** The degree of hemispheric CSE conversion in SCI patients with diseases courses in the range of 0-6 m and 6-36 m. The ln(DH/NDH ratio) were -0.05 ± 0.1 and -0.07 ± 0.2 in 0-6 m and 6-36 m groups, respectively, indicating a decreased CSE of the DH M1 area in SCI patients. *p<0.05, **p<0.01. MEP: motor evoked potentials; DH: dominant hemisphere; NDH: non-dominant hemisphere; CMCT: central motor conduction time; M1 hand area MEP: upper extremity MEP; CST: corticospinal tract; CSE: corticospinal excitability.

To observe the CSE changes in the bilateral M1 hand areas after SCI, the bilateral M1 hand area MEP were compared between biMEP patients and healthy controls. Results showed that the DH M1 hand area MEP amplitude was significantly larger than the NDH M1 hand area MEP amplitude in healthy controls (*p <* 0.01), whereas in SCI patients, the opposite phenomenon appeared, and the NDH M1 hand area MEP amplitude was larger than the DH M1 hand area MEP amplitude. Moreover, the bilateral M1 hand area MEP amplitude in healthy controls were larger than those measured in SCI patients (DH: *p <* 0.01; NDH: *p <* 0.05; Figure 3A). Furthermore, the M1 hand area MEP latency results showed that the bilateral latency of SCI patients were more delayed than those of healthy controls (*p <* 0.01; Figure 3B). No significant difference was found in the M1 hand area MEP CMCT results (Figure 3C).

Considering that the larger M1 hand area MEP amplitude was observed on the DH side of the control, while in SCI, the larger amplitude was observed on the NDH side. The appearance of this opposite trend, led us to analyze the M1 hand area MEP amplitude hemispheric differences, the results from which showed that the ln (DH/NDH ratio) was 0.41 ± 0.1 in the controls, but −0.06 ± 0.1 in the SCI patients. Furthermore, in SCI patients the CSE of the DH M1 hand area decreased and was lower than that of the NDH, as compared with controls (Figure 3D). Based on the finding of previous studies that the majority of motor recovery and AIS conversion occurred within the first 6 months after SCI (20, 27, 28), we divided patients into a 0–6 m and 6–36 m group to further analyze the effects of disease course. SCI patients with a disease course of 0–6 m and 6–36 m had an ln (DH/NDH ratio) of −0.05 ± 0.1 and − 0.07 ± 0.2, respectively, both of which were lower than that of the controls (0–6 m vs. controls: *p <* 0.01, 6–36 m vs. controls: *p <* 0.05; Figure 3E).

Collectively, these results indicated that, in healthy controls, the CSE of the DH M1 area was greater than that of the NDH, whereas in SCI patients, the opposite phenomenon, named hemispheric CSE conversion, was observed in which the CSE of the DH M1 hand area was significantly decreased.

### 3.3. Hemispheric CSE conversion was correlated with motor function and ADL ability in the different SCI subgroups

The correlation of hemispheric CSE conversion with motor function/ADL ability was further analyzed in the different SCI subgroups.

#### 3.3.1. Disease course

**Figure 4:**
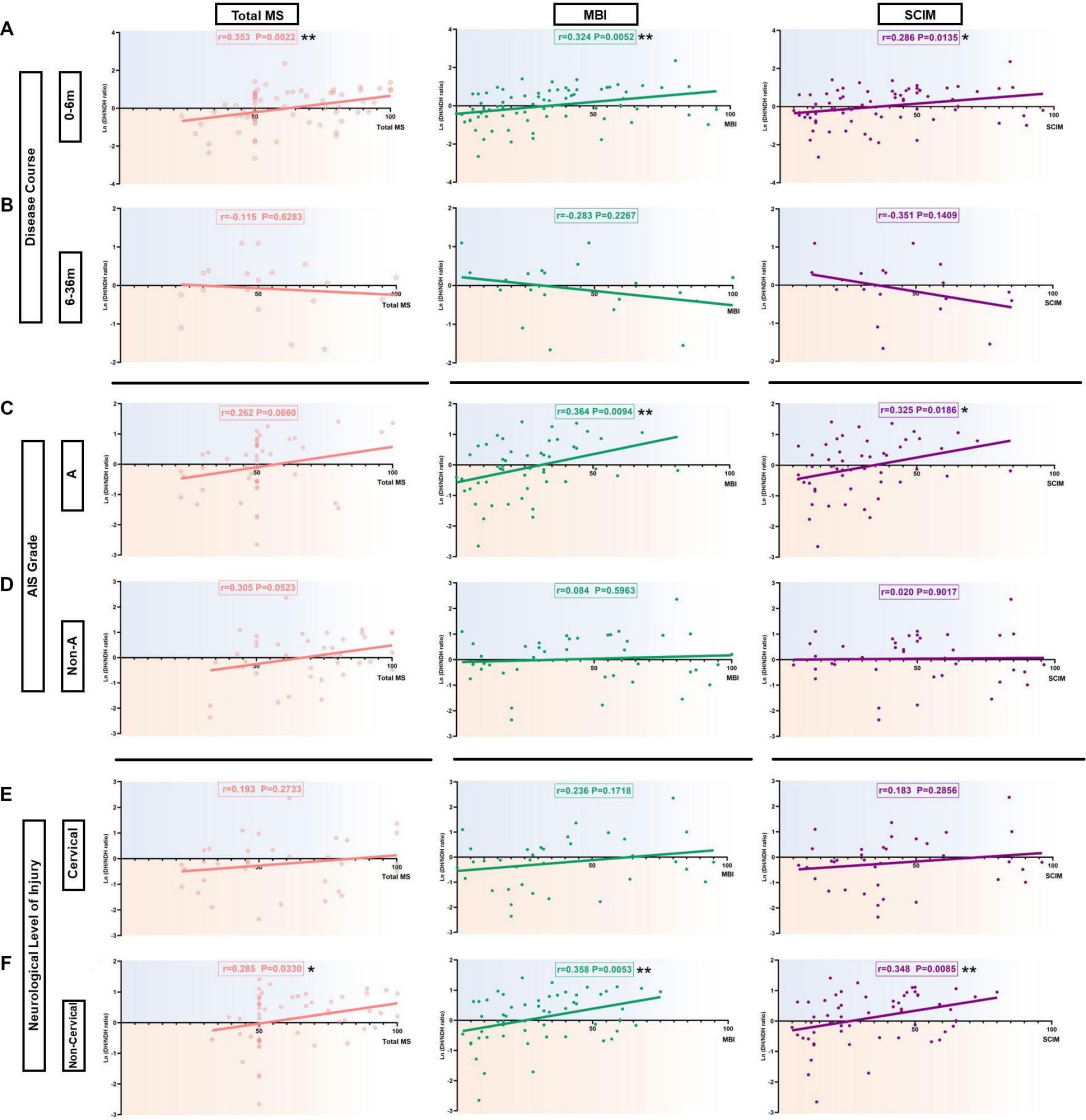
Correlation of the hemispheric CSE conversion degree with total MS and ADL ability in SCI subgroup patients. **(A)** Correlation of the hemispheric CSE conversion degree with total MS and ADL ability in the 0-6 m group. There was a significant positive correlation with the ln(DH/NDH ratio) and total MS (*r*=0.353), MBI (*r*=0.324), as well as SCIM (*r*=0.286). **(B)** Correlation of the hemispheric CSE conversion degree with total MS and ADL ability in the 6-36 m group. There was a negative correlation with the ln(DH/NDH ratio) and total MS (*r*=-0.115), MBI (*r*=-0.283), as well as SCIM (*r*=-0.351), but with no significance. **(C)** Correlation of the hemispheric CSE conversion degree with total MS and ADL ability in the AIS A group. In total MS, we observed that the ln(DH/NDH ratio) had a positive correlation that almost reached significance (*r*=0.262). There was a significant positive correlation with the ln(DH/NDH ratio) and MBI (*r*=0.364) and SCIM (*r*=0.325). **(D)** Correlation of the hemispheric CSE conversion degree with total MS and ADL ability in the AIS Non-A group. In total MS, we observed that the ln(DH/NDH ratio) had a positive correlation trend with no significance (*r*=0.305), and no correlation with MBI (*r*=0.084) or SCIM (*r*=0.020). **(E)** Correlation of the hemispheric CSE conversion degree with total MS and ADL ability in the cervical injury group. The ln(DH/NDH ratio) had no correlation with total MS (*r*=0.193), MBI (*r*=0.236), or SCIM (*r*=0.183). **(F)** Correlation of the hemispheric CSE conversion degree with total MS and ADL ability in the non-cervical injury group. The ln(DH/NDH ratio) was positively correlated with total MS (*r*=0.285), MBI (*r*=0.358), and SCIM (*r*=0.348). *p<0.05, **p<0.01. ADL: activities of daily living; DH: dominant hemisphere; NDH: non-dominant hemisphere; SCIM: spinal cord independence measure; MBI: modified Barthel index; MS: motor score; AIS: American Spinal Injury Association (ASIA) impairment scale; CSE: corticospinal excitability.

In the 0–6 m group, the results showed a significant positive correlation of the ln (DH/NDH ratio) with total MS (*r* = 0.353, *p <* 0.01), MBI (*r* = 0.324, *p <* 0.01), and SCIM (*r* = 0.286, *p <* 0.05; Figure 4A). When further analysis was applied to the UEMS and LEMS, results showed a significant positive correlation of the ln (DH/NDH ratio) with UEMS (*r* = 0.329, *p <* 0.05). Interestingly, the ln (DH/NDH ratio) was also significantly positively correlated with LEMS (*r* = 0.250, *p <* 0.05), indicating the relevance of M1 hand area MEP and LE motor function (Supplementary Figures S1A,C). SCIM12 (*r* = 0.144, *p =* 0.2289) and MAS (*r* = −0.048, *p =* 0.6833) showed no significant correlation with the ln (DH/NDH ratio) (Supplementary Figures S1E,G).

In the 6–36 m group, results showed the opposite trend, but with no significance. The ln (DH/NDH ratio) was negatively correlated with total MS (*r* = −0.115, *p =* 0.6283), MBI (*r* = −0.283, *p =* 0.2267), and SCIM (*r* = −0.351, *p =* 0.1409; Figure 4B). A similar tendency was observed in the results of SCIM12 (*r* = −0.351, *p =* 0.1409) (Supplementary Figure S1F). No significant correlation was found between the ln (DH/NDH ratio) and UEMS (*r* = −0.060, *p =* 0.8014), LEMS (*r* = −0.119, *p =* 0.6171), or MAS (*r* = −0.122, *p =* 0.5899) (Supplementary Figures S1B,D,H).

#### 3.3.2. AIS grade

To assess the effect of degree of spinal cord injury in hemispheric CSE conversion, the AIS A group and the AIS Non-A (including AIS B/C/D) group were further analyzed.

In the AIS A group, the ln (DH/NDH ratio) was significantly positively correlated with MBI (*r* = 0.364, *p <* 0.01) and SCIM (*r* = 0.325, *p <* 0.05), and was positively correlated with total MS, almost reaching significance (*r* = 0.262, *p* = 0.0660; Figure 4C). The ln (DH/NDH ratio) was also significantly positively correlated with LEMS (*r* = 0.298, *p <* 0.05; Supplementary Figure S2C). There was no significant correlation of UEMS (*r* = 0.187, *p =* 0.1934), SCIM12 (*r* = 0.122, *p =* 0.3932), or MAS (*r* = −0.132, *p =* 0.3457) with ln (DH/NDH ratio; Supplementary Figures S2A,E,G).

In the AIS Non-A group, the results showed only a marginal positive correlation of the ln (DH/NDH ratio) with total MS (*r* = 0.305, *p* = 0.0523; Figure 4D). The ln (DH/NDH ratio) had no correlation with MBI (*r* = 0.084, *p =* 0.5963) or SCIM (*r* = 0.020, *p =* 0.9017; Figure 4D), and further analysis indicated there was no correlation of ln (DH/NDH ratio) with UEMS (*r* = 0.142, *p* = 0.3707), LEMS (*r* = 0.272, *p =* 0.0816), SCIM12 (*r* = 0.064, *p =* 0.7026), or MAS (*r* = 0.138, *p =* 0.3654; Supplementary Figures S2B,D,F,H).

#### 3.3.3. NLI

Considering that the lesion of cervical spinal injury might interfere with the measurement of the M1 hand area MEP amplitude stability, SCI patients were divided into a cervical injury and a non-cervical injury (including T/L/S) group for further analysis.

In the cervical injury group, no correlation was found between the ln (DH/NDH ratio) and total MS (*r* = 0.193, *p* = 0.2733), MBI (*r* = 0.236, *p =* 0.1718), or SCIM (*r* = 0.183, *p =* 0.2856; Figure 4E). Moreover, there was no correlation of the ln (DH/NDH ratio) with UEMS (*r* = 0.039, *p =* 0.8284), LEMS (*r* = 0.231, *p =* 0.1896), SCIM12 (*r* = 0.172, *p =* 0.3376), or MAS (*r* = −0.036, *p =* 0.8308; Supplementary Figures S3A,C,E,G).

In the non-cervical injury group, the results showed a significant positive correlation between the ln (DH/NDH ratio) and total MS (*r* = 0.285, *p <* 0.05), MBI (*r* = 0.358, *p <* 0.01) as well as SCIM (*r* = 0.348, *p <* 0.01; Figure 4F). Further analysis of the total MS indicated that the ln (DH/NDH ratio) was also significantly positively correlated with LEMS (*r* = 0.286, *p <* 0.05; Supplementary Figure S3D). The SCIM12 result (*r* = 0.241, *p* = 0.0796) showed a similar tendency (Supplementary Figure S3F), whereas, the ln (DH/NDH ratio) had no correlation with UEMS (*r* = 0.013, *p* = 0.9218) or MAS (*r* = 0.024, *p =* 0.8531) (Supplementary Figures S3B,H).

### 3.4. Hemispheric CSE conversion contributed to changes in ADL ability

To adjusting the possible confounding factors, we utilized MBI and SCIM as the analysis outcomes and conducted a univariate and multivariate linear regression analysis.

For MBI, the results of the univariate analysis showed that the degree of hemispheric CSE conversion, NLI, AIS grade, and education all contributed to outcomes (*p <* 0.10). When conducting the multivariate analysis based on these variables subsequently, the results showed that the degree of hemispheric CSE conversion (unstandardized *β* = 6.067, *p =* 0.023) and AIS grade (D vs. A: unstandardized *β* = 21.483, *p <* 0.001) contributed significantly to outcomes (Table 3).

**Table 3:** Linear regression analysis with MBI and SCIM.

Variable	SCI patients	Univariate analysis (MBI)		Multivariate analysis (MBI)		Univariate analysis (SCIM)		Multivariate analysis (SCIM)	
		Unstandardized β (95% CI)	*P*	Unstandardized β (95% CI)	*P*	Unstandardized β (95% CI)	*P*	Unstandardized β (95% CI)	*P*
Age in years (Mean ± SE)	40.9 ± 1.4	0.176 (−0.199, 0.551)	0.354	–		0.169 (−0.189, 0.527)	0.35	–	
Gender [N (%)]									
Female	21 (20.6)	–		–		–		–	
Male	81 (79.4)	4.335 (−8.205, 16.875)	0.494			7.908 (−3.642, 19.457)	0.177		
SCI type [N (%)]									
Non-traumatic	20 (19.6)	–		–		–		–	
Traumatic	82 (80.4)	−3.91 (−16.756, 8.935)	0.547			−2.7 (−14.855, 9.456)	0.66		
Hemispheric CSE conversion degree (Mean ± SE)	−0.06 ± 0.1	6.867 (1.667, 12.068)	**0.01**	6.067 (0.854, 11.279)	**0.023**	5.638 (0.56, 10.717)	**0.03**	5.597 (0.916, 10.278)	**0.02**
Disease course									
0–6 m	80 (78.4)	–		–		–		–	
6–36 m	22 (21.6)	0.794 (−12.078, 13.665)	0.903			2.686 (−9.76, 15.132)	0.669		
NLI [N (%)]									
C	39 (38.2)	–		–		–		–	
T	40 (39.2)	−9.464 (−20.356, 1.427)	**0.088**	−6.035 (−16.552, 4.482)	0.257	−10.738 (−21.005, −0.471)	**0.041**	−7.262 (−17.036, 2.512)	0.143
L	18 (17.6)	−1.514 (−16.121, 13.093)	0.837	1.548 (−13.748, 16.845)	0.841	−2.528 (−16.432, 11.376)	0.719	−0.802 (−14.780, 13.177)	0.909
S	5 (5.0)	5.914 (−18.465, 30.294)	0.631	3.859 (−18.352, 26.07)	0.73	13.472 (−13.055, 39.999)	0.316	15.642 (−8.099, 39.382)	0.194
AIS grade [N (%)]									
A	54 (52.9)	–		–		–		–	
B	7 (6.9)	10.939 (−6.375, 28.252)	0.213	6.452 (−12.084, 24.988)	0.49	12.423 (−4.013, 28.859)	0.137	10.766 (−6.026, 27.559)	0.206
C	13 (12.7)	8.653 (−5.643, 22.949)	0.232	8.957 (−5.423, 23.336)	0.219	11.137 (−2.419, 24.693)	0.106	11.448 (−1.719, 24.615)	0.087
D	28 (27.5)	23.349 (12.518, 34.179)	**<0.001**	21.483 (10.211, 32.756)	**<0.001**	24.546 (14.145, 34.947)	**<0.001**	22.404 (11.809, 32.999)	**<0.001**
Education [N (%)]									
Less than primary school	2 (1.9)	−27.5 (−62.518, 7.518)	0.122	−21.646 (−53.871, 10.578)	0.185	−23.038 (−57.087, 11.011)	0.182	–	
Completed primary school	17 (16.7)	−11.929 (−29.686,5.829)	0.185	−8.436 (−25.006, 8.134)	0.314	−10.472 (−27.458, 6.515)	0.224		
Less than high school	45 (44.1)	−13.333 (−27.966, 1.299)	**0.074**	−11.152 (−24.852, 2.549)	0.109	−10.469 (−24.657, 3.72)	0.146		
Completed high school	21 (20.6)	−5.158 (−21.752, 11.436)	0.538	−8.987 (−24.167, 6,192)	0.242	−1.316 (−17.632, 15.000)	0.873		
Some/Completed college	17 (16.7)	–		–		–			

Traumatic SCI is defined as an SCI lesion caused by external injury, including heavy object compression, traffic accidents, and falls. Non-traumatic SCI is defined as an SCI lesion caused by other causes, such as congenital conditions or disease processes, including tumors, intervertebral disk compression, and other diseases (43).

SCI, spinal cord injury; ADL, activities of daily living; MEP, motor evoked potentials; MBI, modified Barthel index; AIS, American Spinal Injury Association (ASIA) impairment scale; NLI, neurological level of injury; CSE, corticospinal excitability. Bold values indicates statistical differences of *p*-value.

For SCIM, the results of the univariate analysis showed that the degree of hemispheric CSE conversion, NLI, and AIS grade contributed to outcomes (*p <* 0.10). When conducting the multivariate analysis based on these variables subsequently, the results showed that the degree of hemispheric CSE conversion (unstandardized *β* = 5.597, *p =* 0.020) and AIS grade (D vs. A: unstandardized *β* = 22.404, *p <* 0.001) contributed significantly to outcomes (Table 3).

In conclusion, the above results confirmed that the degree of hemispheric CSE conversion is an independent factor for assessing changes in ADL ability.

### 3.5. Hemispheric CSE conversion conformed more with 0–6 m, AIS-A grade, and non-cervical injury SCI patients

**Figure 5:**
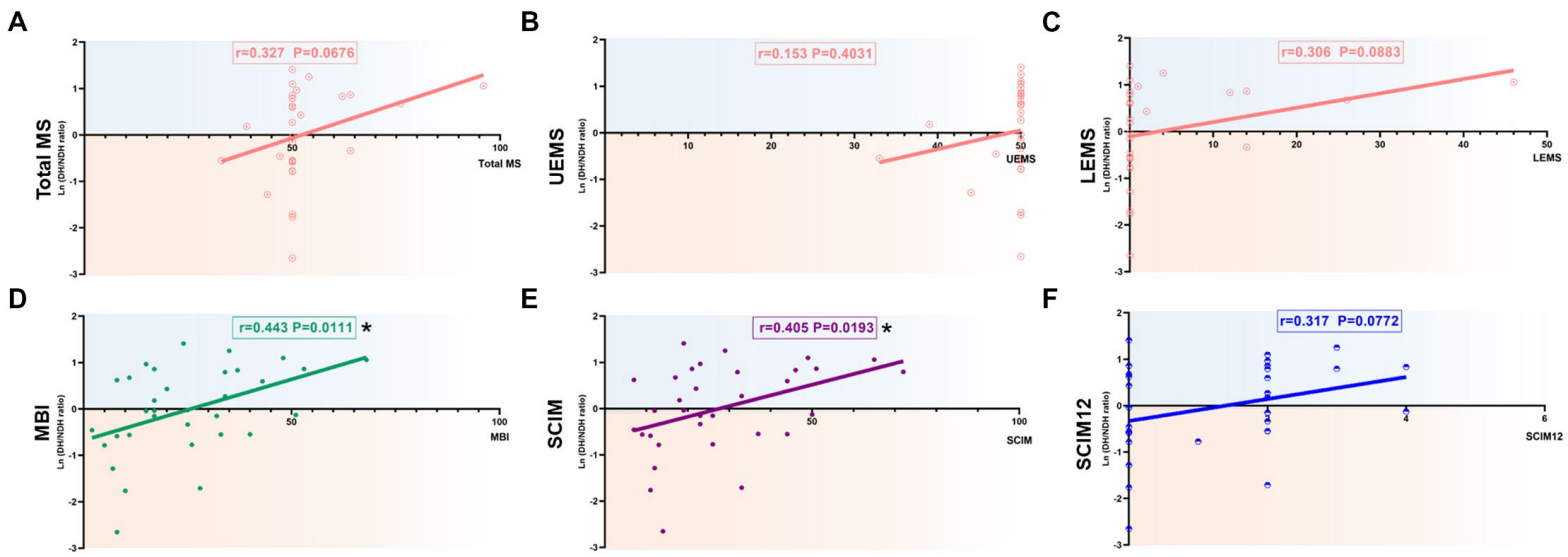
Correlation of the degree of hemispheric CSE conversion with extremity motor function and ADL ability in 0-6 m, AIS-A, and non-cervical injury SCI patients. **(A-C)** The ln(DH/NDH ratio) was positively correlated with total MS (*r*=0.327) and LEMS (*r*=0.306), but had no correlation with UEMS (*r*=0.153). **(D)** There was a significant positive correlation with the ln(DH/NDH ratio) and MBI (*r*=0.443). **(E,F)** There was a significant positive correlation with the ln(DH/NDH ratio) and SCIM (*r*=0.405), and a positive correlation with SCIM12 (*r*=0.317). *p<0.05. ADL: activities of daily living; SCI: spinal cord injury; ADL: activities of daily living; MEP: motor evoked potentials; DH: dominant hemisphere; NDH: non-dominant hemisphere; SCIM: spinal cord independence measure; MBI: modified Barthel index; AIS: American Spinal Injury Association (ASIA) impairment scale; CSE: corticospinal excitability.

SCI patients with 0–6 m, AIS-A grade, and non-cervical injury were selected as the targeted patient group for further analysis. The results of correlation analysis of total MS (*r* = 0.327, *p =* 0.06), LEMS (*r* = 0.306, *p =* 0.0883), and SCIM12 (*r* = 0.317, *p =* 0.0772) showed a positive correlation with the ln (DH/NDH ratio), almost reaching significance (Figures 5A,C,F). Meanwhile, the results of MBI (*r* = 0.443, *p <* 0.05) and SCIM (*r* = 0.405, *p <* 0.05) showed a significant positive correlation with the ln (DH/NDH ratio; Figures 5D,E). The correlation coefficient has increased compared with the above subgroup analysis results. Despite this, the ln (DH/NDH ratio) had no correlation with UEMS (*r* = 0.153, *p* = 0.4031; Figure 5B).

Univariate analysis with MBI showed that the degree of hemispheric CSE conversion and education contributed to outcomes (*p <* 0.10). Multivariate analysis based on these variables showed that the degree of hemispheric CSE conversion (unstandardized *β* = 7.482, *p =* 0.014) contributed to outcomes significantly (Table 4). Univariate analysis with SCIM showed that only the degree of hemispheric CSE conversion (unstandardized *β* = 7.495, *p =* 0.015) contributed to outcomes significantly (Table 4). Unstandardized β values have also increased 1.078 (MBI, univariate analysis), 1.415 (MBI, multivariate analysis), and 1.857 (SCIM, univariate analysis), compared with the above subgroup analysis results.

**Table 4:** Linear regression analysis with MBI and SCIM (0–6 m, AIS A grade, and non-cervial injury SCI patients).

Variable	SCI patients	Univariate analysis (MBI)		Multivariate analysis (MBI)		Univariate analysis (SCIM)	
		Unstandardized β (95% CI)	*P*	Unstandardized β (95% CI)	*P*	Unstandardized β (95% CI)	*P*
Age in years (Mean ± SE)	42.6 ± 1.9	0.217 (−0.452, 0.887)	0.51	–		0.378 (−0.269, 1.024)	0.241
Gender [N (%)]							
Female	4 (12,5)	–		–		–	
Male	28 (87.5)	11.905 (−8.142, 31.952)	0.234			9.893 (−8.572, 28.358)	0.283
SCI type [N (%)]							
Non-traumatic	4 (12.5)	–		–		–	
Traumatic	28 (87.5)	6 (−14.424, 26.424)	0.553			3.607 (−15.175, 22.389)	0.698
Hemispheric CSE conversion degree (Mean ± SE)	−0.02 ± 0.2	7.945 (2.333, 13.558)	**0.007**	7.482 (1.643, 13.321)	**0.014**	7.495 (1.561, 13.429)	**0.015**
Education [N (%)]							
Less than primary school	1 (3.1)	−18.667 (−56.98, 19.647)	0.326	−13.289 (−48.164, 21.586)	0.44	−20.333 (−60.364, 19.697)	0.307
Completed primary school	5 (15.6)	−17.867 (−42.098, 6.365)	0.142	−16.647 (−38.565, 5.270)	0.13	−8.333 (−33.651, 16.984)	0.505
Less than high school	17 (53.1)	−20.292 (−41.167, 0.584)	**0.056**	−15.584 (−34.802, 3.634)	0.107	−17.804 (−39.514, 3.906)	0.104
Completed high school	6 (18.8)	−22.167 (−45.629, 1.295)	**0.063**	−20.25 (−41.504, 1.003)	0.061	−20.167 (−44.680, 4.347)	0.103
Some/Completed college	3 (9.4)	–		–		–	

Traumatic SCI is defined as an SCI lesion caused by external injury, including heavy object compression, traffic accidents, and falls. Non-traumatic SCI is defined as an SCI lesion caused by other causes, such as congenital conditions or disease processes, including tumors, intervertebral disk compression, and other diseases (43).

SCI, spinal cord injury; ADL, activities of daily living; MEP, motor evoked potentials; MBI, modified Barthel index; AIS, American Spinal Injury Association (ASIA) impairment scale; NLI, neurological level of injury; CSE, corticospinal excitability. Bold values indicates statistical differences of *p*-value.

We can thus conclude that, in targeted SCI patients, the degree of hemispheric CSE conversion was more positively correlated with extremities motor function/ADL ability, suggesting that hemispheric CSE conversion conformed more with targeted SCI patients.

## 4. Discussion

Our research has suggested that SCI patients exhibit decreased CSE of the DH M1 hand area. Focusing on the bilateral M1 hand area MEP amplitude, we found that M1 hand area MEP hemispheric CSE conversion was correlated with ADL ability and extremity motor function, especially LE motor function, in SCI patients. Multiple linear regression analysis also confirmed that the degree of M1 hand area MEP hemispheric CSE conversion contributed to changes in ADL ability as an independent factor following confounding adjustment. A positive correlation between the degree of hemispheric CSE conversion and extremity motor function/ADL ability could be found in the 0–6 m, AIS-A grade, and non-cervical injury SCI patients, which indicated that the closer the degree of M1 hand area MEP hemispheric conversion is to that of the healthy controls, the better the overall functional recovery patients can achieve.

### 4.1. Bilateral M1 hand area MEP reflects the CSE changes underlining SCI

Focusing on bilateral M1 hand area MEP between SCI patients and controls, we found that the bilateral M1 hand area MEP latency of SCI patients was more delayed than that of controls, accordant with other reports, which found that a 2–8 ms delay in the MEP latency when comparing SCI patients and controls (29, 30). For MEP CMCT, no significant change was found. As the vital metric for evaluating the integrity of the hemisphere CST (31), our results indicated that the nerve conduction pathways of SCI patients were essentially normal. From this perspective, the MEP differences between patients and controls could be attributed more to the M1 area and CST functional changes.

For MEP amplitude, first, our results showed that the bilateral M1 hand area MEP amplitude in the controls was larger than that of patients, and even in SCI patients with LE MD, the amplitude of M1 hand area MEP was greatly reduced. The MEP amplitude provides insight toward sensitivity to parameter changes for each muscle group (32) and reveals the connection between CST transmission and motor behavior. In this view, MEP amplitude is an excellent means to evaluate motor recovery. Second, the bilateral MEP amplitude comparison showed an opposite phenomenon between patients and controls. The right-hand controls exhibited the larger M1 hand area MEP amplitude of the DH over that of the NDH, revealing the hemispheric asymmetry phenomenon. This hemispheric asymmetry has been widely reported from functional (33) to structural (34) aspects. In humans, hemispheric differences were found in the motor system and electrophysiological assessment (35). Ridding MC et al. confirmed a low-level short-interval intracortical inhibition (SICI) in the DH, indicating the high excitability of the DH (35). The MEP results of the controls in our report reflected the high CSE of the DH M1 hand area, in agreement with the research detailed above.

Contrastingly, an opposite phenomenon was found in SCI patients, showing a larger M1 hand area MEP amplitude of the NDH than that of the DH. In CNS diseases, Mundorf et al. summarized that alterations in hemispheric asymmetry are widespread across almost all disorders (34). In this view, the MEP hemispheric conversion of SCI patients suggested that the bilateral M1 hand area and the CST might have undergone functional changes. Several studies have demonstrated this phenomenon, including that of Freund et al., which revealed the reduced fractional anisotropy in the CST of the left M1 hand-knob area in SCI patients compared with healthy controls using diffusion-tensor imaging metrics (36). Therefore, our M1 hand area MEP hemispheric conversion results most likely revealed a decreased CSE of the bilateral M1 hand area in SCI patients, and was decreased to a greater degree in the DH side.

### 4.2. The degree of hemispheric CSE conversion might serve as a potential indicator of overall functional recovery

The changes in motor function were analyzed from the aspects of MS and SCIM12. The analysis of the total MS showed the same trend with ADL ability in the subgroup divided by disease course, AIS grade, and NLI. When analyzing UEMS and LEMS separately, we found the relevance of LEMS and M1 hand area MEP, in other words, the degree of M1 hand area MEP hemispheric CSE conversion could inform on the changes in LE motor function. The results of SCIM12 further confirmed the relevance of M1 hand area MEP and LE motor function, as SCIM12 evaluates SCI patient indoor mobility function (24). The results of SCIM12 showed a similar tendency with LEMS, that the degree of M1 hand area MEP hemispheric CSE conversion was positively correlated with SCIM12 in 0–6 m, AIS A grade, non-cervical injury patients. Previous research in *Cell* revealed that the M1 hand area of SCI patients encodes the activity information of both the UE and LE, and that the movement coding of the UE and LE are highly correlated (8). In this view, the present research supports the opinion that the M1 hand area and LE motor function are highly correlated. The evaluation of the degree of M1 hand area MEP hemispheric CSE conversion may assess changes in extremity motor function, especially LE motor function.

Apart from motor function, we further observed changes in ADL ability. We analyzed two ADL assessment scales for mutual verification. The results suggested that the analysis of MBI and SCIM had good consistency.

Interestingly, our findings showed a totally different correlation tendency in SCI subgroup patients. First, in the patients with a disease course of 0–6 m, the degree of hemispheric CSE conversion was positively correlated with ADL ability. However, the opposite trend was found in 6–36 m patients, where the degree of hemispheric CSE conversion was negatively correlated with ADL ability, indicating that corticospinal plasticity might begin to fulfill an important role with the extension of disease course. Several studies have demonstrated CST or cortical reorganization following SCI. Oudega and Perez reported that electrophysiological studies have provided evidence for corticospinal reorganization, which may contribute to functional recovery after SCI (37). Urbin et al. also summarized the occurrence of significant reorganization of motor maps after SCI (4). These studies have revealed the large capacity for cortical or CST plasticity that can be reflected in the MEP changes after SCI (11). From this perspective, cortical or CST plasticity might begin to fulfill a leading role during the first 6 months of the disease course, which may be suitable timing for SCI patient therapeutic intervention (20). Steeves et al. demonstrated a typical rapid recovery pattern in motor scores before 6 months, but that slows down from 6 months to 1 year (27). Waters et al. also revealed that the rate of motor recovery rapidly declined in the first 6 months and then approached a plateau (28). Fawcett et al. summarized that the most rapid motor recovery improvement occurs within the first 6 months after SCI, and neurological recovery will also incline to a stable baseline after 6 months (38). Accordingly, our results strongly suggested that 6 months was the demarcation point of recovery, but that the exact mechanism still requires further exploration.

Second, in the patients with AIS A grade, the degree of hemispheric CSE conversion was significantly positively correlated with ADL ability, while no correlation was observed in AIS non-A patients. We speculated that more pronounced and complicated cortical or CST changes are present in incomplete SCI (AIS non-A grade), so as to induce changes that differ from the above results that “the closer the degree of M1 hand area hemispheric CSE conversion is to healthy controls, the better ADL ability patients get.” Similar to our results, Wirth et al. saw no relationship between MEP amplitudes and recovery of ambulation and muscle strength in incomplete SCI patients. The author explained that this phenomenon might be attributed to the plastic changes in spinal neural circuits and preserved motor units (39).

Third, in the patients with different NLI, results indicated that patients in the non-cervical injury group had a significantly positive correlation with degree of hemispheric CSE conversion and ADL ability, while a positive correlation without significance was found in the cervical injury group. This finding may due to the influence of cervical injury on the UE CST pathway.

### 4.3. Regulating bilateral M1 hand areas excitability may be a novel strategy for SCI rehabilitation

Multivariate linear regression analysis suggested that the degree of hemispheric CSE conversion and AIS grade contributed to ADL changes. Apart from the degree of hemispheric CSE conversion, our linear regression analysis of AIS grade was in agreement with previous research that AIS D grade patients showed higher ADL ability than AIS A grade patients (40). Meanwhile, it is worth noting that analysis of the level of neurological injury showed no significant differences. This is probably due to the circumstance that M1 hand area MEP cannot be measured in some cervical injury SCI patients who were included in an aMEP or uniMEP group, and the remaining cervical injury SCI patients in the biMEP group with only mild symptoms were included in the linear regression analysis (only biMEP group patients could calculate degree of MEP hemispheric conversion).

In the analysis of targeted patients (0–6 m, AIS A grade, and non-cervical injury), our results revealed that the degree of hemispheric CSE conversion was positively correlated with ADL ability, total MS, and LEMS. The correlation coefficient increased compared with the above subgroup analysis results performed separately. Linear regression analysis showed that degree of hemispheric CSE conversion affected outcomes significantly, and that the unstandardized β increased at the same time. From this aspect, the targeted SCI patients conformed more with the law that “the closer the degree of M1 hand area MEP hemispheric CSE conversion is to that of healthy controls, the better the extremity motor function/ADL ability a patient will get.” In this view, targeted regulation to the M1 hand area excitability might improve the motor function and ADL ability in SCI patients. Our study may provide the instructive guidance for standardized therapeutic intervention in the future, that is, to enhance the NDH M1 hand area excitability but attenuate the DH M1 hand area excitability, so as to regulate the degree of hemispheric CSE conversion in the SCI patient closer to that of normal status.

Our research does have some limitations. Although the total number of included patients reached 320, only one-third of patients had complete, analyzable, bilateral M1 hand area MEP data. Therefore, the number of patients (including paraplegia and conus injury patients) still needs to be expanded continuously to verify the degree of hemispheric CSE conversion. Additionally, our research is a retrospective study, a cohort study is urgently needed to observe the relationship between hemispheric CSE conversion and extremity motor function/ADL ability. Based on the law of this phenomenon in SCI patients, targeted intervention like rTMS on the M1 hand area should also be conducted subsequently through a randomized controlled trial. In this study, we utilized MEP to evaluate hemispheric excitability conversion in SCI patients, which reflected the excitability changes of both the CST and M1 area. In terms of intracortical excitability changes, multi-level brain measurements, including motor map (41) based on Brodmann area, paired-pulse TMS, and functional near-infrared spectroscopy (42) should also be considered.

In conclusion, our study has revealed that the CSE of the DH M1 hand area decreased in SCI patients. Furthermore, the M1 hand area MEP hemispheric CSE conversion was independently correlated with the extremity motor function/ADL ability of SCI patients, which might serve as a potential indicator for overall functional recovery. In the targeted SCI patients, the closer the degree of the M1 hand area MEP hemispheric CSE conversion was to that of healthy controls, the better the extremity motor function/ADL ability patients achieve. Based on this phenomenon, targeted intervention to regulate the excitability of bilateral M1 hand areas to normal status might be a novel strategy for the overall functional recovery of SCI patients. Besides, the CSE conversion may serve as a potential indicator for the evaluation of functional prognosis with the ultimate goal to improve the quality of life in SCI patients.

## Data availability statement

The original contributions presented in the study are included in the article/Supplementary material, further inquiries can be directed to the corresponding authors.

## Ethics statement

This study was approved by the Ethics Review committee of the Xijing Hospital (No. KY20222073-C-1). The ethics committee waived the requirement of written informed consent for participation.

## Author contributions

HY and X-LS designed the manuscript. C-QD and MG wrote the report. X-DL, B-JX, YL, and XH conducted the statistical analysis. M-LX, X-BW, G-QC, and C-GZ participated in figures drawing. All authors read and approved the final manuscript.

## Funding

This work was supported by grants from the National Natural Science Foundation of China (82272591 and 82072534).

## Conflict of interest

The authors declare that the research was conducted in the absence of any commercial or financial relationships that could be construed as a potential conflict of interest.

## Publisher’s note

All claims expressed in this article are solely those of the authors and do not necessarily represent those of their affiliated organizations, or those of the publisher, the editors and the reviewers. Any product that may be evaluated in this article, or claim that may be made by its manufacturer, is not guaranteed or endorsed by the publisher.

## Abbreviations

SCI: spinal cord injury
rTMS: repeated transcranial magnetic stimulation ADL activities of daily living
MD: motor dysfunction M1 motor cortex MEP motor evoked potentials
DH: dominant hemisphere
NDH: non-dominant hemisphere
UEMS: upper extremity motor score
LEMS: lower extremity motor score
AIS: American Spinal Injury Association (ASIA) impairment scale
MAS: modified Ashworth scale
MBI: modified Barthel index
SCIM: spinal cord independence measure
SEM: standard error of the mean
NLI: neurological level of injury
CMCT: central motor conduction time
aMEP: abolished MEP group
uniMEP: unilateral MEP group
biMEP: bilateral MEP group
SICI: short-interval intracortical inhibition
CST: corticospinal tract
CSE: corticospinal excitability
MAP-2: microtubule-associated protein 2
BDNF: brain-derived neurotrophic factor

## References

[1] Singh A Tetreault L Kalsi-Ryan S Nouri A Fehlings MG. Global prevalence and incidence of traumatic spinal cord injury. Clin Epidemiol. (2014) 6:309–31. doi: 10.2147/CLEP.S68889, PMID: 25278785 PMC4179833

[2] Alizadeh A Dyck SM Karimi-Abdolrezaee S. Traumatic spinal cord injury: an overview of pathophysiology, models and acute injury mechanisms. Front Neurol. (2019) 10:282. doi: 10.3389/fneur.2019.00282, PMID: 30967837 PMC6439316

[3] Solstrand Dahlberg L Becerra L Borsook D Linnman C. Brain changes after spinal cord injury, a quantitative meta-analysis and review. Neurosci Biobehav Rev. (2018) 90:272–93. doi: 10.1016/j.neubiorev.2018.04.018 29702136

[4] Urbin MA Royston DA Weber DJ Boninger ML Collinger JL. What is the functional relevance of reorganization in primary motor cortex after spinal cord injury? Neurobiol Dis. (2019) 121:286–95. doi: 10.1016/j.nbd.2018.09.009, PMID: 30217521

[5] Krogh S Aagaard P Jønsson AB Figlewski K Kasch H. Effects of repetitive transcranial magnetic stimulation on recovery in lower limb muscle strength and gait function following spinal cord injury: a randomized controlled trial. Spinal Cord. (2022) 60:135–41. doi: 10.1038/s41393-021-00703-8, PMID: 34504284 PMC8428490

[6] Rossi S Antal A Bestmann S Bikson M Brewer C Brockmöller J. Safety and recommendations for TMS use in healthy subjects and patient populations, with updates on training, ethical and regulatory issues: expert guidelines. Clin Neurophysiol. (2021) 132:269–306. doi: 10.1016/j.clinph.2020.10.003, PMID: 33243615 PMC9094636

[7] Lefaucheur JP Aleman A Baeken C Benninger DH Brunelin J di Lazzaro V. Evidence-based guidelines on the therapeutic use of repetitive transcranial magnetic stimulation (rTMS): an update (2014-2018). Clin Neurophysiol. (2020) 131:474–528. doi: 10.1016/j.clinph.2019.11.002, PMID: 31901449

[8] Willett FR Deo DR Avansino DT Rezaii P Hochberg LR Henderson JM. Hand knob area of premotor cortex represents the whole body in a compositional way. Cells. (2020) 181:396–409.e26. doi: 10.1016/j.cell.2020.02.043, PMID: 32220308 PMC7166199

[9] Belci M Catley M Husain M Frankel HL Davey NJ. Magnetic brain stimulation can improve clinical outcome in incomplete spinal cord injured patients. Spinal Cord. (2004) 42:417–9. doi: 10.1038/sj.sc.3101613, PMID: 15111994

[10] Gunduz A Rothwell J Vidal J Kumru H. Non-invasive brain stimulation to promote motor and functional recovery following spinal cord injury. Neural Regen Res. (2017) 12:1933–8. doi: 10.4103/1673-5374.221143, PMID: 29323025 PMC5784334

[11] Bestmann S Krakauer JW. The uses and interpretations of the motor-evoked potential for understanding behaviour. Exp Brain Res. (2015) 233:679–89. doi: 10.1007/s00221-014-4183-7 25563496

[12] Hendricks HT van Limbeek J Geurts AC Zwarts MJ. Motor recovery after stroke: a systematic review of the literature. Arch Phys Med Rehabil. (2002) 83:1629–37. doi: 10.1053/apmr.2002.35473 12422337

[13] Sheng W Li S Zhao J Wang Y Luo Z Lo WLA. Upper limbs muscle co-contraction changes correlated with the impairment of the corticospinal tract in stroke survivors: preliminary evidence from electromyography and motor-evoked potential. Front Neurosci. (2022) 16:886909. doi: 10.3389/fnins.2022.886909, PMID: 35720692 PMC9198335

[14] Oyama K Hu L Sakatani K. Prediction of MMSE score using time-resolved near-infrared spectroscopy. Adv Exp Med Biol. (2018) 1072:145–50. doi: 10.1007/978-3-319-91287-5_23, PMID: 30178337

[15] Ziemann U Reis J Schwenkreis P Rosanova M Strafella A Badawy R. TMS and drugs revisited 2014. Clin Neurophysiol. (2015) 126:1847–68. doi: 10.1016/j.clinph.2014.08.028, PMID: 25534482

[16] Klein MM Treister R Raij T Pascual-Leone A Park L Nurmikko T. Transcranial magnetic stimulation of the brain: guidelines for pain treatment research. Pain. (2015) 156:1601–14. doi: 10.1097/j.pain.0000000000000210, PMID: 25919472 PMC4545735

[17] Jutzeler CR Streijger F Aguilar J Shortt K Manouchehri N Okon E. Sensorimotor plasticity after spinal cord injury: a longitudinal and translational study. Ann Clin Transl Neurol. (2019) 6:68–82. doi: 10.1002/acn3.679, PMID: 30656185 PMC6331953

[18] Long JY Federico P Perez MA. A novel cortical target to enhance hand motor output in humans with spinal cord injury. Brain. (2017) 140:1619–32. doi: 10.1093/brain/awx102, PMID: 28549131 PMC6059088

[19] Poole BJ Mather M Livesey EJ Harris IM Harris JA. Motor-evoked potentials reveal functional differences between dominant and non-dominant motor cortices during response preparation. Cortex. (2018) 103:1–12. doi: 10.1016/j.cortex.2018.02.004, PMID: 29533856

[20] Kirshblum S Snider B Eren F Guest J. Characterizing natural recovery after traumatic spinal cord injury. J Neurotrauma. (2021) 38:1267–84. doi: 10.1089/neu.2020.7473, PMID: 33339474 PMC8080912

[21] Meseguer-Henarejos AB Sánchez-Meca J López-Pina JA Carles-Hernández R. Inter- and intra-rater reliability of the modified Ashworth scale: a systematic review and meta-analysis. Eur J Phys Rehabil Med. (2018) 54:576–90. doi: 10.23736/S1973-9087.17.04796-7, PMID: 28901119

[22] Prodinger B Oâ€™Connor R Stucki G Tennant A. Establishing score equivalence of the functional independence measure motor scale and the Barthel index, utilising the international classification of functioning, disability and health and Rasch measurement theory. J Rehabil Med. (2017) 49:416–22. doi: 10.2340/16501977-2225 28471470

[23] Kapadia N Masani K Catharine Craven B Giangregorio LM Hitzig SL Richards K. A randomized trial of functional electrical stimulation for walking in incomplete spinal cord injury: effects on walking competency. J Spinal Cord Med. (2014) 37:511–24. doi: 10.1179/2045772314Y.0000000263, PMID: 25229735 PMC4166186

[24] van Middendorp JJ Hosman AJF Donders ART Pouw MH Ditunno JF Jr Curt A. A clinical prediction rule for ambulation outcomes after traumatic spinal cord injury: a longitudinal cohort study. Lancet. (2011) 377:1004–10. doi: 10.1016/S0140-6736 (10)62276-3 21377202

[25] Vaalto S Säisänen L Könönen M Julkunen P Hukkanen T Määttä S. Corticospinal output and cortical excitation-inhibition balance in distal hand muscle representations in nonprimary motor area. Hum Brain Mapp. (2011) 32:1692–703. doi: 10.1002/hbm.21137, PMID: 20886574 10.1002/hbm.21137 PMC6870022

[26] Petersen JA Spiess M Curt A Dietz V Schubert M EM-SCI Study Group. Spinal cord injury: one-year evolution of motor-evoked potentials and recovery of leg motor function in 255 patients. Neurorehabil Neural Repair. (2012) 26:939–48. doi: 10.1177/1545968312438437 22460611

[27] Steeves JD Kramer JK Fawcett JW Cragg J Lammertse DP Blight AR. Extent of spontaneous motor recovery after traumatic cervical sensorimotor complete spinal cord injury. Spinal Cord. (2011) 49:257–65. doi: 10.1038/sc.2010.99, PMID: 20714334

[28] Waters RL Adkins RH Yakura JS Sie I. Motor and sensory recovery following complete tetraplegia. Arch Phys Med Rehabil. (1993) 74:242–7. 8439249

[29] Smith HC Savic G Frankel HL Ellaway PH Maskill DW Jamous MA. Corticospinal function studied over time following incomplete spinal cord injury. Spinal Cord. (2000) 38:292–300. doi: 10.1038/sj.sc.3100994, PMID: 10822402

[30] Cariga P Catley M Nowicky AV Savic G H Ellaway P Davey NJ. Segmental recording of cortical motor evoked potentials from thoracic paravertebral myotomes in complete spinal cord injury. Spine (Phila Pa 1976). (2002) 27:1438–43. doi: 10.1097/00007632-200207010-00013, PMID: 12131743

[31] Hoonhorst MHJ Nijland RHM Emmelot CH Kollen BJ Kwakkel G. TMS-induced central motor conduction time at the non-infarcted hemisphere is associated with spontaneous motor recovery of the paretic upper limb after severe stroke. Brain Sci. (2021) 11:648. doi: 10.3390/brainsci11050648 34063558 PMC8157217

[32] Journee HL Berends HI Kruyt MC. The percentage of amplitude decrease warning criteria for transcranial MEP monitoring. J Clin Neurophysiol. (2017) 34:22–31. doi: 10.1097/WNP.0000000000000338, PMID: 28045854

[33] Gunturkun O Strockens F Ocklenburg S. Brain lateralization: a comparative perspective. Physiol Rev. (2020) 100:1019–63. doi: 10.1152/physrev.00006.2019, PMID: 32233912

[34] Mundorf A Peterburs J Ocklenburg S. Asymmetry in the central nervous system: a clinical neuroscience perspective. Front Syst Neurosci. (2021) 15:733898. doi: 10.3389/fnsys.2021.733898, PMID: 34970125 PMC8712556

[35] Ridding MC Flavel SC. Induction of plasticity in the dominant and non-dominant motor cortices of humans. Exp Brain Res. (2006) 171:551–7. doi: 10.1007/s00221-005-0309-2, PMID: 16501966

[36] Freund P Wheeler-Kingshott CA Nagy Z Gorgoraptis N Weiskopf N Friston K. Axonal integrity predicts cortical reorganisation following cervical injury. J Neurol Neurosurg Psychiatry. (2012) 83:629–37. doi: 10.1136/jnnp-2011-301875, PMID: 22492214 PMC3348614

[37] Oudega M Perez MA. Corticospinal reorganization after spinal cord injury. J Physiol. (2012) 590:3647–63. doi: 10.1113/jphysiol.2012.233189, PMID: 22586214 PMC3476625

[38] Fawcett JW Curt A Steeves JD Coleman WP Tuszynski MH Lammertse D. Guidelines for the conduct of clinical trials for spinal cord injury as developed by the ICCP panel: spontaneous recovery after spinal cord injury and statistical power needed for therapeutic clinical trials. Spinal Cord. (2007) 45:190–205. doi: 10.1038/sj.sc.3102007, PMID: 17179973

[39] Wirth B Van Hedel HJ Curt A. Changes in corticospinal function and ankle motor control during recovery from incomplete spinal cord injury. J Neurotrauma. (2008) 25:467–78. doi: 10.1089/neu.2007.0472, PMID: 18419251

[40] Scivoletto G Farchi S Laurenza L Tamburella F Molinari M. Impact of multiple injuries on functional and neurological outcomes of patients with spinal cord injury. Scand J Trauma Resusc Emerg Med. (2013) 21:42. doi: 10.1186/1757-7241-21-42, PMID: 23718823 PMC3669625

[41] Krause P Forderreuther S Straube A. TMS motor cortical brain mapping in patients with complex regional pain syndrome type I. Clin Neurophysiol. (2006) 117:169–76. doi: 10.1016/j.clinph.2005.09.012, PMID: 16326140

[42] Zhou G Chen Y Wang X Wei H Huang Q Li L. The correlations between kinematic profiles and cerebral hemodynamics suggest changes of motor coordination in single and bilateral finger movement. Front Hum Neurosci. (2022) 16:957364. doi: 10.3389/fnhum.2022.957364, PMID: 36061505 PMC9433536

[43] Shepherd J Tu K Young J Chishtie J Craven BC Moineddin R. Identifying cases of spinal cord injury or disease in a primary care electronic medical record database. J Spinal Cord Med. (2021) 44:S28–39. doi: 10.1080/10790268.2021.1971357, PMID: 34779726 PMC8604482

