# Supplementary material for: Primary motor hand area corticospinal excitability indicates overall functional recovery after spinal cord injury
Source: Front Neurol. 2023 Jun 2;14:1175078. doi: 10.3389/fneur.2023.1175078 (PMC10273270; doi:10.3389/fneur.2023.1175078)
Supplement: Supplementary file 1 [file Data_Sheet_1.docx]

**
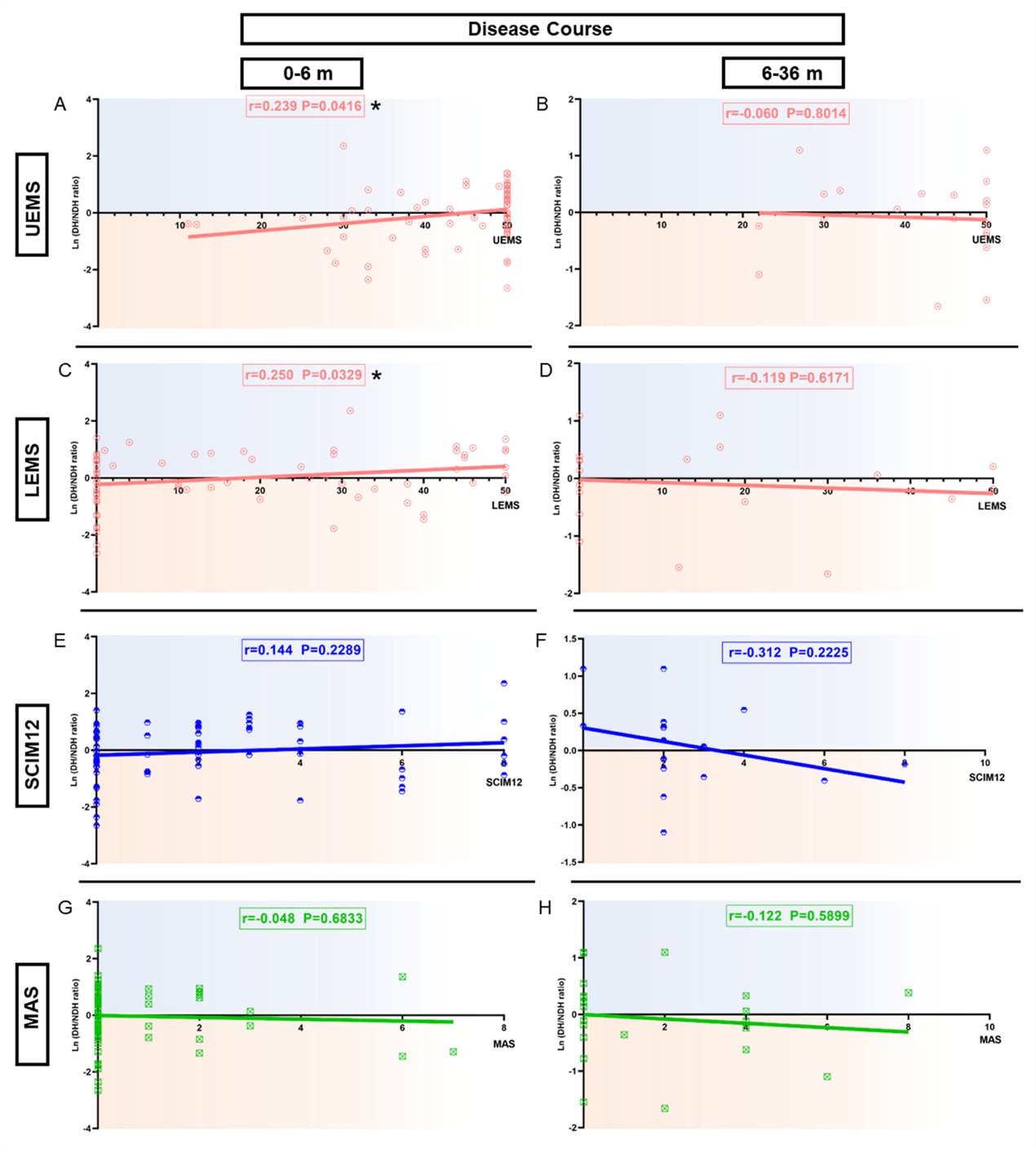
**

**Supplementary Figure 1 Correlation of the degree of M1 hand area MEP hemispheric excitability conversion with UEMS, LEMS, SCIM12, and MAS in SCI patients grouped by disease course.**

**A, C, E and G** Correlation of the degree of M1 hand area MEP hemispheric excitability conversion with UEMS, LEMS, SCIM12, and MAS in the 0-6 m group. The ln (DH/NDH ratio) were significantly positively correlated with UEMS (r=0.329) and LEMS (r=0.250).

**B, D, F and H** Correlation of the degree of M1 hand area MEP hemispheric excitability conversion with UEMS, LEMS, SCIM12, and MAS in the 6-36 m group. The ln (DH/NDH ratio) were negatively correlated with SCIM12 (r=-0.351), but with no significance.

**p<*0.05. Abbreviations: DH: dominant hemisphere; NDH: non-dominant hemisphere; SCIM: spinal cord independence measure; M1 hand area MEP: upper extremity MEP; UEMS: upper extremity motor score; LEMS: lower extremity motor score; MAS: modified Ashworth scale


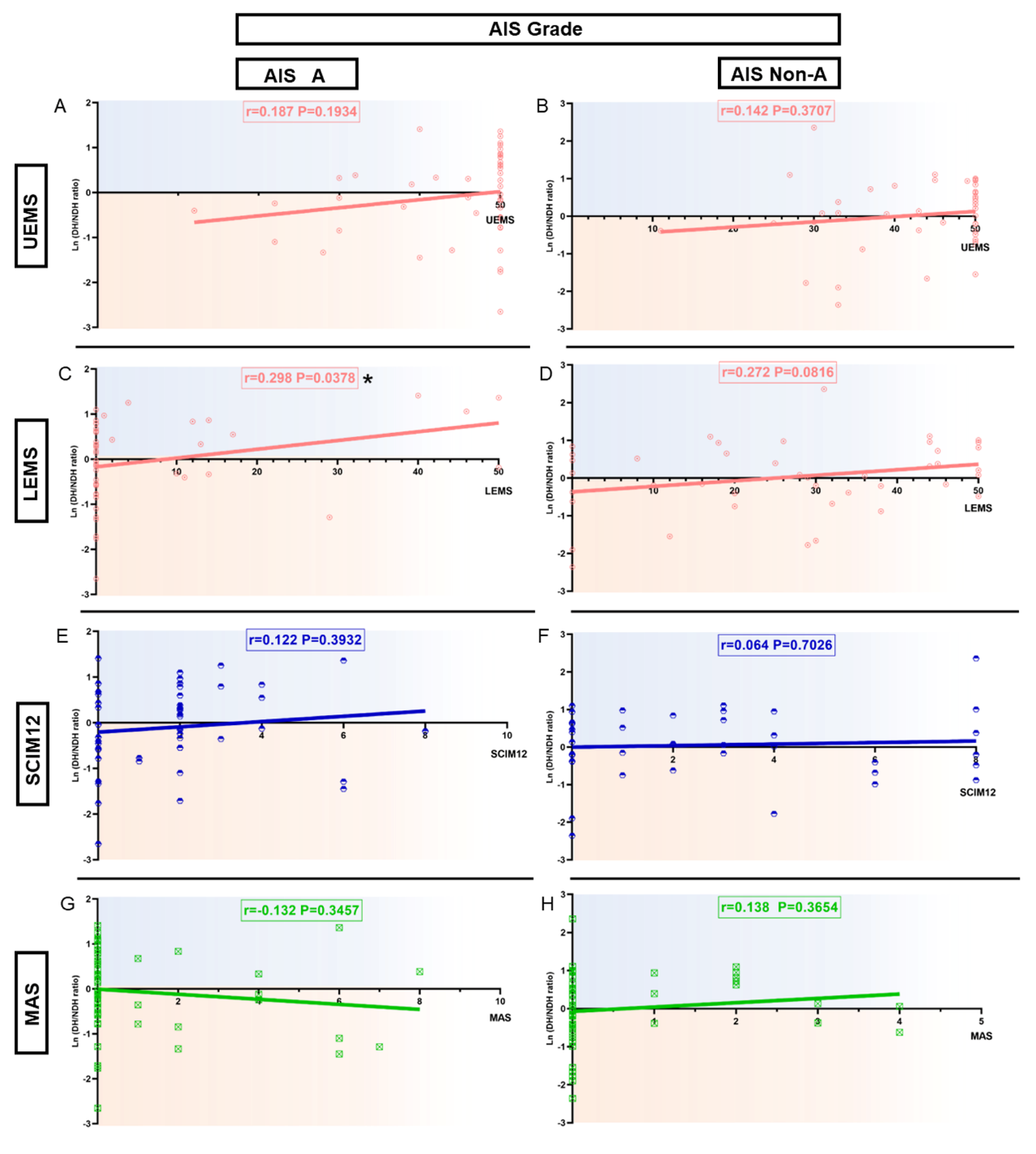


**Supplementary Figure 2 Correlation of the degree of M1 hand area MEP hemispheric excitability conversion with UEMS, LEMS, SCIM12, and MAS in SCI patients grouped by AIS grade.**

**A, C, E and G** Correlation of the degree of M1 hand area MEP hemispheric excitability conversion with UEMS, LEMS, SCIM12, and MAS in the AIS A group. The ln (DH/NDH ratio) were significant positively correlated with LEMS (r=0.298).

**B, D, F and H** Correlation of the degree of M1 hand area MEP hemispheric excitability conversion with UEMS, LEMS, SCIM12, and MAS in the AIS Non-A group. No significant correlation could be found.

**p<*0.05. Abbreviations: DH: dominant hemisphere; NDH: non-dominant hemisphere; AIS: American Spinal Injury Association (ASIA) impairment scale; SCIM: spinal cord independence measure; M1 hand area MEP: upper extremity MEP; UEMS: upper extremity motor score; LEMS: lower extremity motor score; MAS: modified Ashworth scale


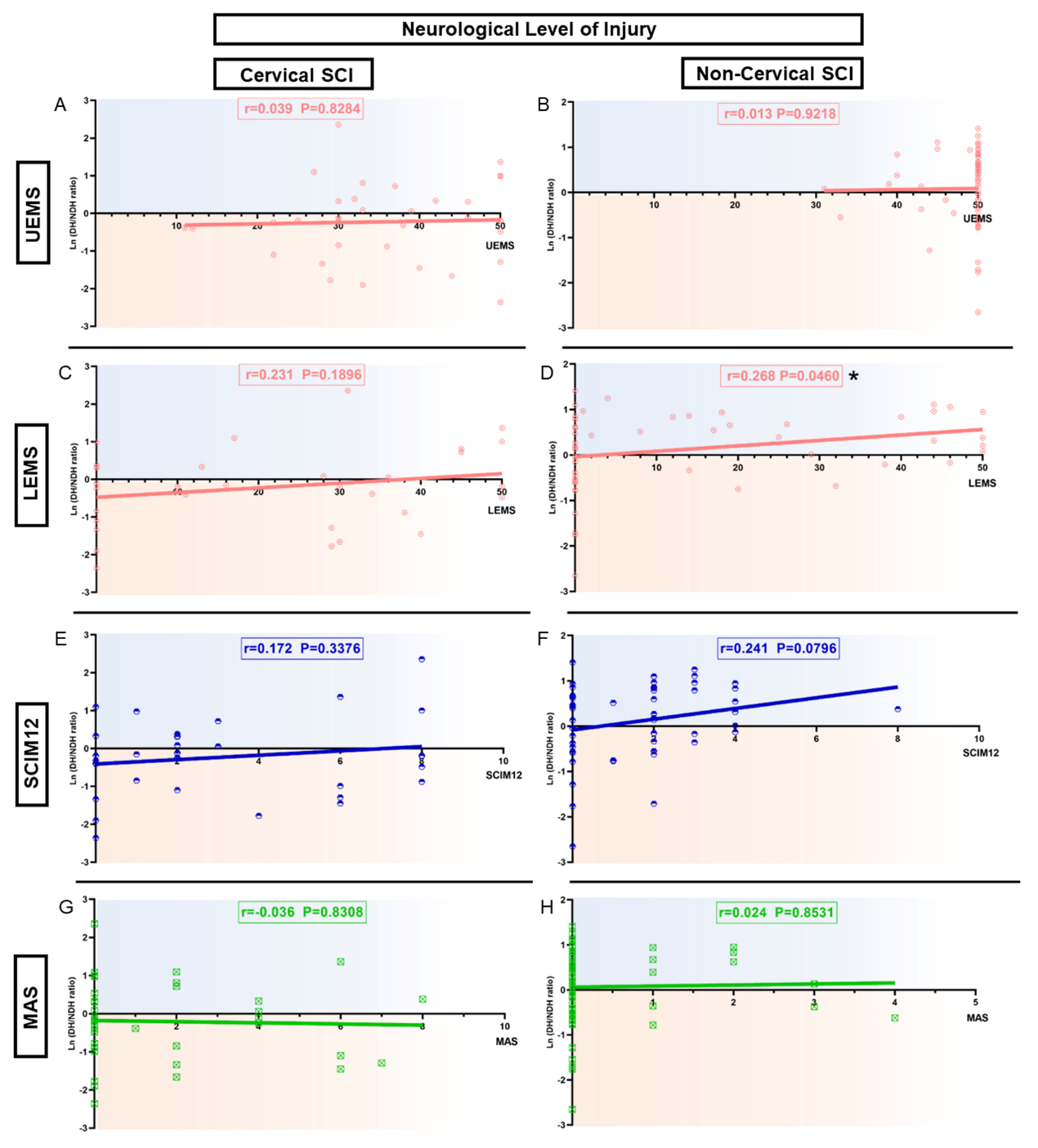


**Supplementary Figure 3 Correlation of the degree of M1 hand area MEP hemispheric excitability conversion with UEMS, LEMS, SCIM12, and MAS in SCI patients grouped by NLI.**

**A, C, E and G** Correlation of the degree of M1 hand area MEP hemispheric excitability conversion with UEMS, LEMS, SCIM12, and MAS in the cervical injury group. No significant correlation could be found.

**B, D, F and H** Correlation of the degree of M1 hand area MEP hemispheric excitability conversion with UEMS, LEMS, SCIM12, and MAS in the non-cervical injury group. The ln (DH/NDH ratio) were significant positively correlated with LEMS (r=0.286). Results of SCIM12 (r=0.241) showed a similar tendency, but with no significance.

**p<*0.05. Abbreviations: SCI: spinal cord injury; DH: dominant hemisphere; NDH: non-dominant hemisphere; SCIM: spinal cord independence measure; M1 hand area MEP: upper extremity MEP; UEMS: upper extremity motor score; LEMS: lower extremity motor score; MAS: modified Ashworth scale
